# Competing with Lower Level Opponents Decreases Intra-Team Movement Synchronization and Time-Motion Demands during Pre-Season Soccer Matches

**DOI:** 10.1371/journal.pone.0097145

**Published:** 2014-05-09

**Authors:** Hugo Folgado, Ricardo Duarte, Orlando Fernandes, Jaime Sampaio

**Affiliations:** 1 Department of Sport and Health, Universidade de Évora, Évora, Portugal; 2 Interdisciplinary Center for the Study of Human Performance (CIPER), Faculdade de Motricidade Humana, Universidade de Lisboa, Lisboa, Portugal; 3 Research Center for Sports Sciences, Health and Human Development (CIDESD), Universidade de Trás-os-Montes e Alto Douro, Vila Real, Portugal; Purdue University, United States of America

## Abstract

This study aimed to quantify the time-motion demands and intra-team movement synchronization during the pre-season matches of a professional soccer team according to the opposition level. Positional data from 20 players were captured during the first half of six pre-season matches of a Portuguese first league team. Time-motion demands were measured by the total distance covered and distance covered at different speed categories. Intra-team coordination was measured by calculating the relative phase of all pairs of outfield players. Afterwards, the percentage of time spent in the −30° to 30° bin (*near-in-phase* mode of coordination) was calculated for each dyad as a measure of space-time movement synchronization. Movement synchronization data were analyzed for the whole team, according to each dyad average speed and by groups of similar dyadic synchronization tendencies. Then, these data were compared according to the opponent team level (first league; second league; amateurs). Time-motion demands showed no differences in total distance covered per opposition levels, while matches opposing teams of superior level revealed more distance covered at very high intensity. Competing against superior level teams implied more time in synchronized behavior for the overall displacements and displacements at higher intensities. These findings suggest that playing against higher-level opponents (1^st^ league teams) increased time-motion demands at high intensities in tandem with intra-team movement synchronization tendencies.

## Introduction

During the pre-season period, professional soccer teams focus on developing both physical fitness and tactical aspects. A common strategy used during this period is to promote friendly preparation matches against opponents of different levels, in addition to the training sessions. However, there is scarce information about the physical and tactical requirements of these preparation matches.

Physical demands of competitive matches have been extensively studied revealing a total distance covered of around 10–12 km by the outfield players mostly by walking and running at low intensities [1], with high intensity running accounting for about 10% of the total distance covered [2]. Mohr et al. [3] studied the seasonal variation of total distance covered and distance covered at high-intensity running during competition, with top-class players having greater results in both variables at the end of the season. Curiously, no differences were found between matches at the beginning and middle of the season. However, some studies have found differences in the fitness of players, as measured by aerobic fitness and sprint speed, between the beginning and middle of the season [4], [5]. This suggests contradictory results between physical performance during the actual match and the players' maximum physiological capabilities. One might speculate that despite being physically fit, players do not operate at their physiological maximum due to the collective game pace imposed to each player [6].

Soccer is a team sport where two opposing teams dynamically interact in order to gain advantage over the other team [7]. In this sense, performance should be understood in terms of space-time interaction dynamics and not only in terms of the players' individual time-motion demands. As such, the analysis of tactical performance in soccer should capture how players individually and collectively adapt to the ever-changing configurations of play, in order to gain advantage over their opponents [8]. Such analysis approach can be based on non-linear dynamical systems theory and uses measures such as relative phase. This measure has enabled the identification of coordinative states in physical, biological and social systems [9]. Several studies have used these techniques to examine player interactions and their relation with performance outcomes, particularly in soccer. For instance, Sampaio and Maçãs [10] used a pre-post test design to assess tactical behaviors in small-sided games, by calculating the relative phase of the distances between each player and their team centroid position. The pre-test values revealed no predominant intra-team coordination mode. However the post-test measurements revealed increased stability towards anti-phase and in-phase modes of coordination, suggesting that stable coordinated movements arise from increased tactical expertise. Also using relative phase analysis, Travassos et al. [11] measured the dyadic intra-team coordination tendencies of futsal (5-a-side indoor soccer) teams. This study showed a strong attraction to in-phase behaviors for the defending team, but a weaker attraction for the attacking team, suggesting that attackers explore various dynamical interactions to disrupt the defensive structure. Additionally, Folgado et al. [12] used the teams' length per width ratio to compare the tactical behavior of young soccer players of different age and expertise levels. The variability of this ratio decreased with increases in the players' age and expertise level. These findings reinforced the notion that a more stable mode of coordination may be linked to the players' increased tactical expertise and subsequent better performance.

Despite the important theoretical and practical contributions promoted by the aforementioned studies, to our knowledge, no research has investigated intra-team coordination combined with time-motion variables in 11-a-side soccer. A related approach [10] using approximate entropy (ApEn) to quantify the regularity of the players' distance to the team center, suggested that players presented a more regular behavior at lower speeds (<13 km · h^−1^), having more difficulty in adjusting their position at higher speeds. As such, we propose that the assessment of the players' movement synchronization tendencies should also consider different speed categories, to allow the understanding of its effects on match performance.

In summary, the findings mentioned above suggest that intra-team measures and time-motion demands might be used to provide complementary insights about contextualized player performance in team sports. In particular, they can reveal how individual and collective performances emerge in the face of different contextual constraints, such as the level of the opponent team. Indeed, the quality of opposition has been proposed as an important factor associated with match performance indicators [13]. For instance, when playing against stronger opposition, a team tends to present less percentage of ball possession [14] and higher distance covered by walking and jogging [15], than when playing against a weaker opponent. Therefore, the aim of the present study was to quantify the time-motion demands and intra-team movement synchronization tendencies of a professional soccer team during the pre-season, according to the level of the opponents. We hypothesize that the level of the opponent team may promote different time-motion demands and that variations in the speed of the players' movement should have a distinct influence in the intra-team synchronization tendencies.

## Methods and Materials

### Participants and data collection

A total of 20 professional players (age = 24.8±3.9 yrs; professional playing experience = 7.1±4.0 yrs) participated in 6 pre-season matches of a Portuguese first league soccer team. Positional data from the outfield players of the analyzed team in each match were collected using 5 Hz GPS units (SPI Pro, GPSports, Canberra, Australia). Previous verifications have established the validity and reliability of this instrumentation (coefficient of variation <5%) [16].

The team faced opponents of different level during the analyzed fixtures, contesting two matches against each opposition level (first league, second league and amateur teams). We only collected positional data from outfield players during each match. Due to the characteristics of the pre-season fixtures, all of the players' in the analyzed team were substituted in the majority of the matches at half time. To ensure a more constant team formation between matches, only the first half of each match was analyzed. During the collected first halves no player was substituted.

Prior to the start of this study, formal authorization was cleared by the club technical staff. Players were instructed about the procedures that would ensue and gave their verbal informed consent to participate in the study. Verbal consent was preferred due to practicality reasons. A research team member documented the players' consent using a checklist, in the presence of an external witness to the study. All procedures were approved by the Ethics Committee of the Research Centre for Sport Sciences, Health and Human Development, based at Vila Real (Portugal).

**Figure 1:**
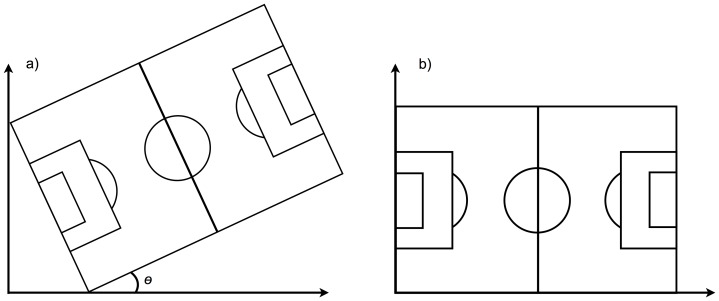
A rotation matrix was applied rotating the data through an angle *θ*. A rotation matrix was calculated from the field vertices and applied to the players' positions, rotating the data through an angle *θ* in order that the longitudinal displacements were aligned with the x-axis and the lateral displacements were aligned with the y-axis.

Positional data were retrieved from GPS units and processed in MATLAB 2011b (The MathWorks Inc., Natick, MA, USA). Latitude and longitude data collected from each individual outfield player were synchronized. Missing data gaps were re-sampled using an interpolation method to guarantee the same length of the time series. Latitude and longitude data were transposed to meters, using the Universal Transverse Mercator (UTM) coordinate system by means of a MATLAB routine [17], and smoothed using a 3 Hz Butterworth low pass filter. After converting the positional data into meters, a rotation matrix was calculated for each match from the field vertices positions, aligning the length of the playing field with the x-axis and the width with the y-axis (Figure 1). The rotation matrix was then applied to the players' positional data for alignment with the playing field referential.

### Time-motion and intra-team synchronization variables

Time-motion variables were the total distance covered by players and distance covered at different movement speed categories (adapted from Carling, 2011 [18]): 0.0–3.5 km · h^−1^ (low intensity); 3.6–14.3 km · h^−1^ (moderate intensity); 14.4–19.7 km · h^−1^ (high intensity); and >19.8 km · h^−1^ (very high intensity).

To assess intra-team coordination tendencies, the relative phase of all pairs of outfield players (n = 45) was calculated to the longitudinal (x-axis) and lateral (y-axis) movement directions in every match, using the Hilbert Transform (for the application of this technique see Palut & Zanone, 2005 [19]). We considered that by sharing a common goal, each pair of teammates could potentially form a dyad, i.e., a pair of two players who share the same environment and intentionality, pursuing common goal-directed behaviors [7]. To quantify the movement synchronization of each dyad, we calculated the percentage of time spent between −30° to 30° of relative phase (*near-in-phase* synchronization mode). This interval was selected based on previous research, which identified in-phase relations between players as the most common mode of coordination [11], [20]. This assumption was also confirmed in our data. Such analysis was first calculated for the overall half and then divided according to each dyad average speed, using the aforementioned movement speed categories.

To classify each dyad into one of three groups according to their synchronization level, a k-means cluster analysis was subsequently applied to the percentage of time of dyadic synchronization. This classification intended to represent a functional clustering method, which captured intra-team dyads with similar levels of synchronization. This method allowed for a more detailed understanding of the hypothesized opposition level effects on different sub-groups of players within the team.

### Statistical analysis

Both time-motion and intra-team synchronization data were considered as dependent variables and compared according to the three levels of opposition (first league, second league and amateurs teams). One-way ANOVA was used to compare time-motion variables and the percentage of time of dyadic synchronization according to opposition level. Synchronization analysis was also divided by dyad average speeds and by cluster classification groups, comparing each group according to the opposing team level using one-way ANOVAs. Effect sizes are presented as partial eta-squared (η^2^). Pairwise comparisons were calculated using Fisher's least significant difference (LSD) tests and Cohen's *d* effect sizes with 95% confidence intervals [21].

Statistical calculations were done using IBM SPSS Statistics (version 20.0, IBM Corporation, Somers, New York, USA) and the package compute.es in R [22]. Statistical significance was maintained at 5%.

## Results

**Figure 2:**
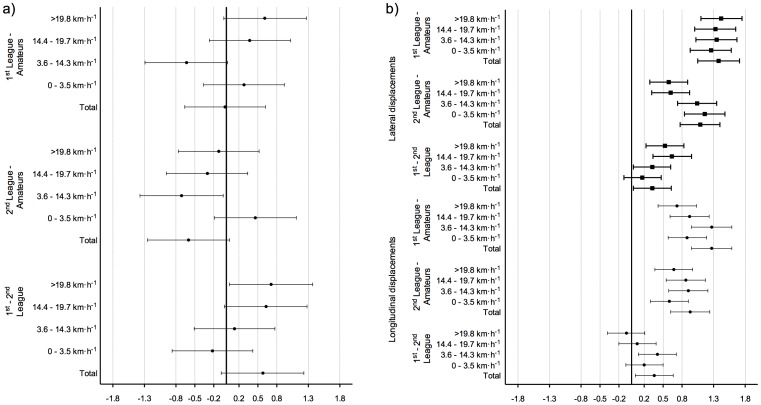
Standardized effect sizes and 95% CI of pairwise differences between opposition levels for time motion (a) and intra-team synchronization (b) variables. Positive values represent superior results in matches opposing the higher-level team.

Time-motion variables showed no differences in the total distance covered between opposition levels (*F*_(2, 57)_ = 2.247, *p* = 0.115, η^2^ = 0.073) (Table 1). An increase in the distance covered at moderate intensity running was observed in matches against amateur players (*F*_(2, 57)_ = 3.425, *p* = 0.039, η^2^ = 0.107). On the other hand, an increase in the distance covered at very high intensity running was also found in matches opposing first league teams (*F*_(2, 57)_ = 3.296, *p* = 0.044, η^2^ = 0.104). Pairwise effect size analyses revealed no clear tendency in time-motion variables between opposition levels (Figure 2).

**Table 1:** Total distance covered (m) and distance covered at several intensities by opposition level.

	Against 1st League (1^st^)	Against 2nd League (2^nd^)	Against amateurs (am)	Pairwise comparison
Total distance covered (m)	5395.3±588.6	5069.7±527.5	5407.9±597.3	
Distance covered at				
Low intensity (0.0–3.5 km **·** h^−1^)	422.2±67.0	436.9±67.2	399.8±91.1	
Moderate intensity (3.6–14.3 km **·** h^−1^)	3655.2±299.5	3615.1±332.3	3896.7±454.3	1^st^<am*; 2^nd^<am*
High intensity (14.4–19.7 km **·** h^−1^)	910.9±306.5	729.3±267.1	807.0±257.1	
Very high intensity (>19.8 km **·** h^−1^)	407.1±193.9	288.4±135.2	304.4±140.2	1^st^>2^nd^, am*

* Significant differences at p<0.05.

**Figure 3:**
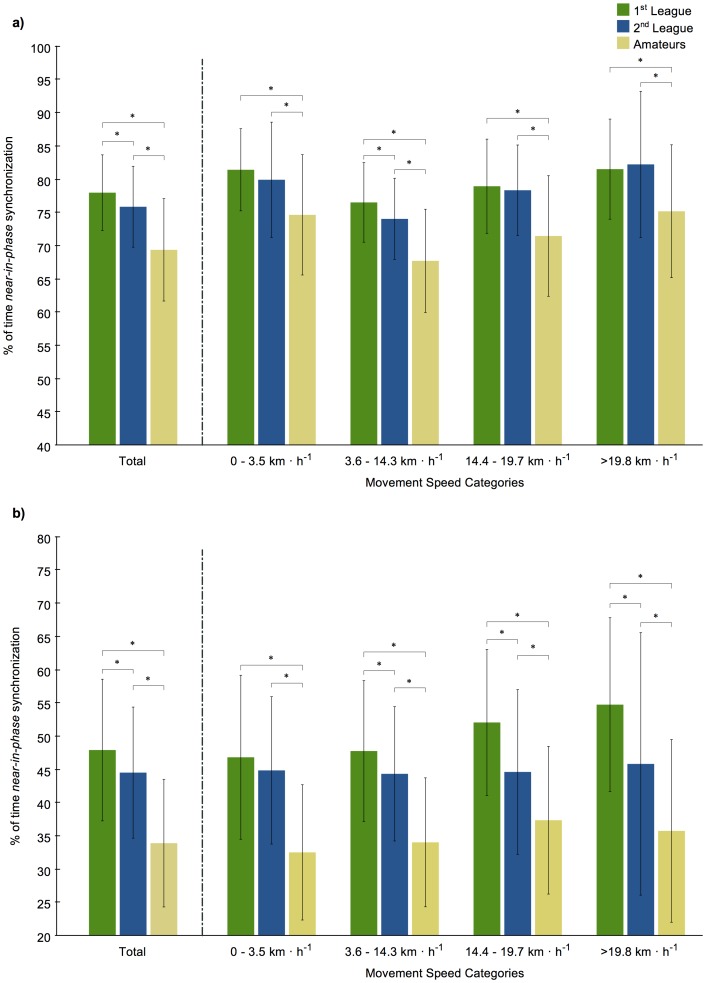
Percentage of time of dyadic synchronization according to the opposition level. a) Longitudinal and b) lateral displacements for the whole analysed half and by different movement speed categories. *: Significant differences at p<0.05.

The overall dyadic movement synchronization tendencies were significantly different according to opposition level in both longitudinal (*F*_(2, 267)_ = 42.149, *p*<0.001, η^2^ = 0.240) and lateral (*F*_(2, 267)_ = 47.626, *p*<0.001, η^2^ = 0.263) displacement axes. Pairwise comparisons showed differences between all opposition levels for both axes, with a higher percentage of time spent in dyadic synchronization in the matches played against higher-level teams (Figure 3). Results also revealed a large effect size for movement synchronization in both axes, when comparing matches against 1^st^ and 2^nd^ league teams with matches against amateur teams (Figure 2).

Movement synchronization data pertaining to each dyad at different average speed categories, revealed significant differences in accordance with the opposing team levels, both for longitudinal (low intensity - *F*_(2, 267)_ = 17.562, *p*<0.001, η^2^ = 0.116; moderate intensity - *F*_(2, 267)_ = 41.555, *p*<0.001, η^2^ = 0.237; high intensity - *F*_(2, 267)_ = 26.080, *p*<0.001, η^2^ = 0.163; very high intensity - *F*_(2, 267)_ = 14.664, *p*<0.001, η^2^ = 0.099) and lateral (low intensity - *F*_(2, 267)_ = 42.858, *p*<0.001, η^2^ = 0.243; moderate intensity - *F*_(2, 267)_ = 44.784, *p*<0.001, η^2^ = 0.251; high intensity - *F*_(2, 267)_ = 36.734, *p*<0.001, η^2^ = 0.216; very high intensity - *F*_(2, 267)_ = 32.501, *p*<0.001, η^2^ = 0.196) displacement axes. Pairwise comparisons also revealed higher percentage of time spent in dyadic synchronization in matches played against higher-level teams. Moderate to large effect sizes were found when comparing matches against 1^st^ and 2^nd^ league teams with matches against amateur teams (Figure 2 and 3).

**Figure 4:**
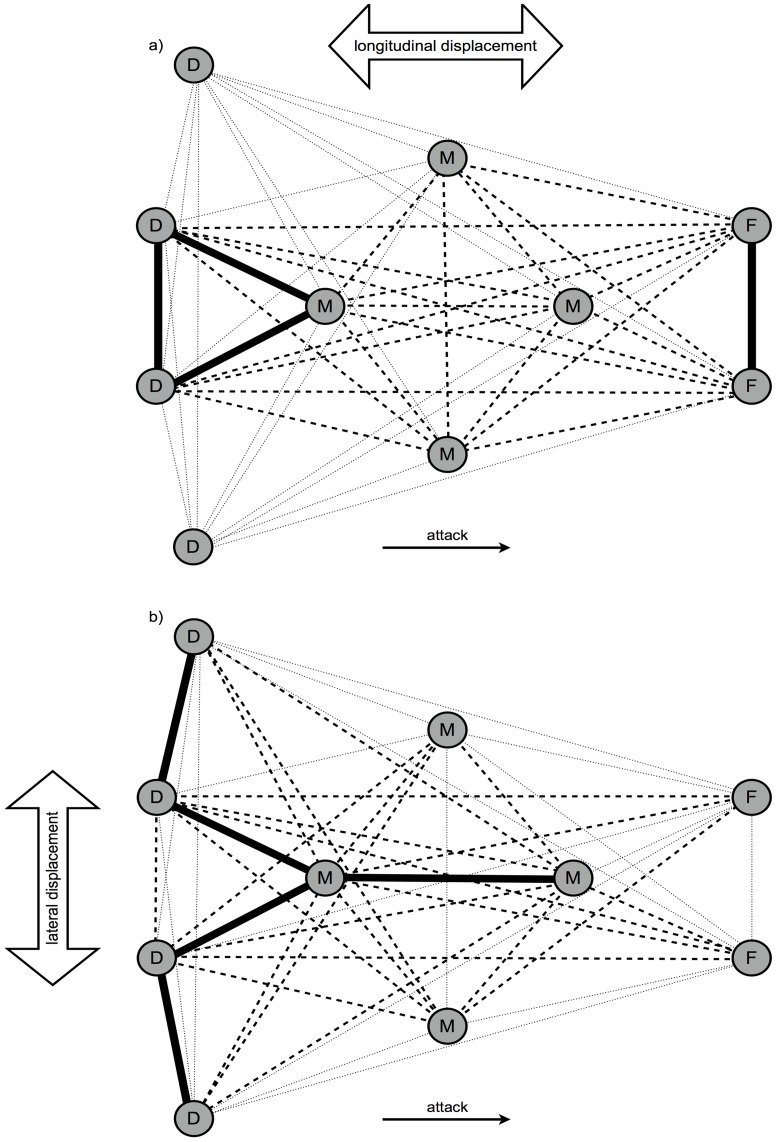
K-means clustering of players' according to the percentage of time of dyadic synchronization. a) Longitudinal and b) lateral displacements of defenders (D), midfielders (M) and forwards (F). Solid lines represent the higher synchronization group; dashed lines represent the intermediate synchronization group; dotted lines represent the low synchronization group.

The k-means cluster analyses allowed for classification of dyads in three different groups for both lateral and longitudinal movements. For the longitudinal direction, the group with higher level of synchronization (86.1%±3.3) was formed by 4 dyads. The group with intermediate level of synchronization (76.3%±2.3) was composed by 22 dyads. Finally, the group with the lowest level of synchronization (69.8%±2.3) was comprised by 19 dyads (Figure 4a). For the lateral direction, the group with higher level of synchronization (58.6%±4.3) was formed by 5 dyads; the intermediate group (44.4%±3.2) comprised 23 dyads; and the lower group (34.2%±4.5) was composed of 17 dyads with (Figure 4b).

**Figure 5:**
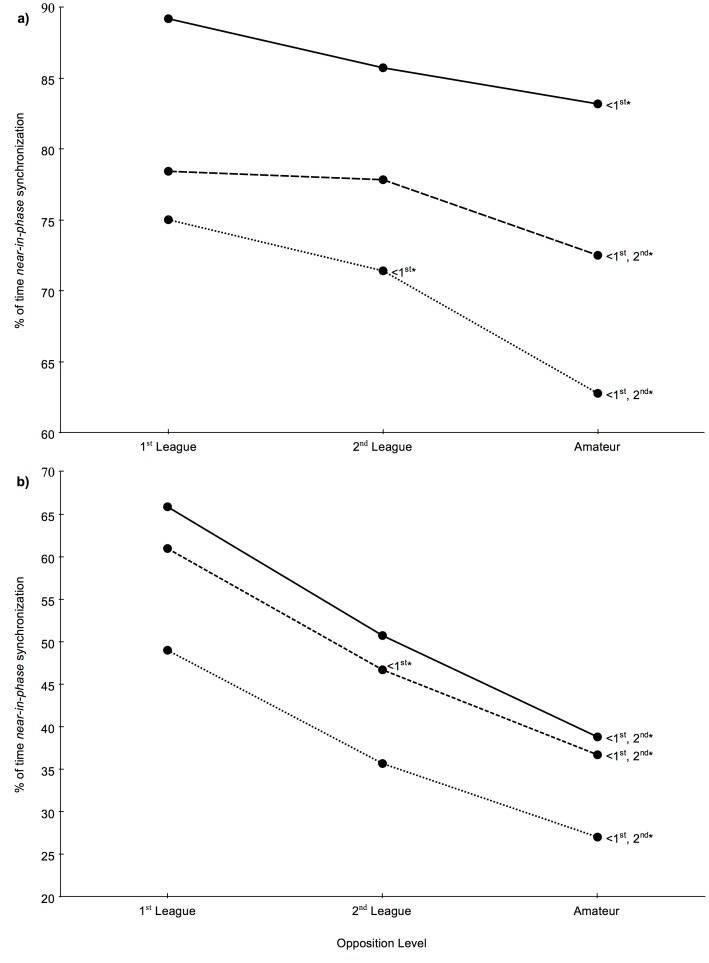
Clustering groups' percentage of time of dyadic synchronization according to the opposition level. a) Longitudinal and b) lateral displacements. Solid lines represent the higher synchronization group; dashed lines represent the intermediate synchronization group; dotted lines represent the low synchronization group. *: Significant differences at p<0.05.

The synchronization data of every cluster revealed significant differences according to the opposing team level both for longitudinal (higher - *F*_(2, 21)_ = 3.894, *p* = 0.036, η^2^ = 0.271; intermediate - *F*_(2, 129)_ = 21.956, *p*<0.001, η^2^ = 0.254; lower - *F*_(2, 111)_ = 102.090, *p*<0.001, η^2^ = 0.648) and lateral (higher - *F*_(2, 27)_ = 11.858, *p*<0.001, η^2^ = 0.468; intermediate - *F*_(2, 135)_ = 66.440, *p*<0.001, η^2^ = 0.496; lower - *F*_(2, 99)_ = 32.021, *p*<0.001, η^2^ = 0.393) displacement axes. Pairwise comparisons showed higher percentage of time spent in synchronization against first league teams than against amateur teams, in all cluster groups in both displacement axes (Figure 5a and b).

## Discussion

The aim of the present study was to quantify the time-motion demands and intra-team movement synchronization tendencies during the pre-season of a professional soccer team, according to the opponent levels. Time-motion analysis showed no differences in the total distance covered between opponent levels, but more distance was covered at very high running intensity, in matches against first league teams. Intra-team movement synchronization was significantly higher when the analyzed professional team faced better level opponents. These differences in movement synchronization presented higher magnitude when matches opposing professional level teams (1^st^ and 2^nd^ league) were compared to matches opposing non-professional amateur teams. In this study, the on-field movement synchronization of players seems to reflect the differences between levels of opposition.

The higher amount of time spent in synchronization when competing against better teams may be explained by the greater demands imposed by higher-level opponents. It is possible that these demands might enhance the need of collaborative work in order to gain advantage over the higher-level opponents, in both attack and defense game phases [23]. Also, the superior level of synchronization reported for the longitudinal direction of the playing field is in line with other studies in team sports, in which opposing teams competed with the same number of players [10], [24], [25]. However, when playing against teams with numerical superiority, a superior level of synchronization was observed in the lateral direction of the playing field [11].

The analysis of movement synchronization data, according to dyad average movement speed, allowed further examination of the influence of the opposition team level. The players tended to be more synchronized at low and very high intensities for longitudinal displacements and at very high intensities for lateral displacements. This association suggests that periods of very high running intensity may be responsible for the global increase of dyadic synchronization that was identified in this study. Therefore, we suggest that game pacing can act as a moderator variable of intra-team synchronization. Interestingly, while the amount of distance covered at some of the movement speed categories did not vary, the players' movement synchronization levels were sensitive enough to discriminate the different levels of opposition in every movement speed category. These findings underline the importance of a coordination measure as complementary and necessary to gain new insights in performance analysis of soccer [26], [27]. In line with this findings, a recent work on small sided soccer games showed evidence of higher irregularity in the way each player coordinated its movements with teammates at fast pace [27]. However, to the best of our knowledge, this is the first attempt to interrelate players' movement synchronization and time motion variables in 11-a-side soccer matches. These recent data, together with the findings presented here, suggest that albeit in a more unpredictable manner, players tend to display high levels of movement synchronization during fast paced moments. Moreover, recent literature suggests that these moments can be critical in match performance, for example during goal-scoring situations [28]. Thus, the preparation of teams during pre-season can potentially benefit from competing with opponents of superior level, which simultaneously increases the physical demands and the intra-team synchronization processes.

Our time-motion findings contradict those of Lago et al. [15], who showed that a higher level of opposition represented a higher amount of distance covered at low intensities. However, their study compared opposing teams within the same league throughout a season and during the competitive phase, while our approach studied teams of very different competitive standards and during the preparatory phase of a season. This aspect may hypothetically affect the results, by amplifying the opposition level differences. Nevertheless, and even during the competitive phase, Rampinini et al. [29] showed time-motion data that agree with the findings of our work, with players covering more distance at high intensity running against the best ranked opponents.

Previous research has shown the diversity of interpersonal coordination tendencies in terms of the strength of attraction that some dyads exhibit within a team (e.g. [10], [24]). In our study, we used k-means cluster analyses to identify the different groups regarding the level of movement synchronization in each displacement axis. For example, the movement synchronization between lateral defenders and central defenders was high for the lateral displacements, but remained lower for the longitudinal displacements. This finding suggests that the coupling of players, at an intra-team level, does not exclusively occur with neighboring players as has been previously suggested in the literature [7]. It is possible that specific goal-directed behaviors pursued at local and global scales should influence the coupling of players [30]. However, further data has also shown that all dyads grouped into higher synchronized clusters were formed by neighboring players. As suggested in the literature [25], [31], this finding may reveal a certain degree of dependence on spatial proximity when it comes to the development of superior levels of movement synchronization, However, it seems that this proximity does not necessarily imply synchrony. From a practical perspective, this classification method may be useful for identifying the interpersonal relations of players and select specific training situations to improve team tactical coordination. As an example, when designing tasks for promoting lateral coordination of the defensive line, the presence of both lateral and center defenders must be considered to enhance the movement synchronization between them. On the other hand, tasks designed to promote longitudinal coordination of the defensive line, must consider the presence of both center defenders and the defensive midfielder.

Despite the intra-team focus of our analysis, our data has shown that the level of the opponent team presents a determinant role in the dyadic coordination of players. This further highlights the adaptive characteristics of the behavior of soccer teams as an emergent process under the influence of multiple interacting performance constraints [30], [32], such as the level of the opponent. A recent study did not find any differences in the level of collective synchronization when considering ball possession [33]. As such, our study did not distinguish the attacking and defending phases during the match. Nevertheless, this distinction could potentially help to explain the higher amount of time spent in movement synchronization that emerges in matches against stronger opponents. Future research might explore this issue further.

The complementary relation between time motion variables and movement synchronization tendencies may also provide useful insights for coaches. Specifically, players tend to spend more time in near synchronized behavior and at higher speeds of movement in matches against stronger opponents. From a practical point of view, coaches can use this information to improve quantitative evaluations of tactical performance and later to design representative practice tasks to enhance transfer from training sessions to the match context [34]. For example, raising the quality of opposition in a training situation may promote not only greater physical impact, but also a more synchronized behavior between players. These adaptations should help optimize the individual and collective behaviors expected to arise during competitions.

## Conclusions

Selecting stronger opponents for matches during the pre-season seems to promote more synchronized behaviors between players and elicit greater physical demands for professional soccer teams. The results also suggest that decreasing the opponent level tends to lower the required movement synchronization. When preparing the pre-season fixtures, teams should be aware that playing against opponents of lower levels might not present sufficient stimulus for tactical and physical development.

The analysis of soccer performance based on the players' positional data can gain from the integration of time-motion and movement synchronization variables. Such integration can provide further insights to the understanding of collaborative teamwork and game dynamics. The matches investigated allow for the speculation that the dyadic synchronization of players may serve as a relevant performance indicator. The cluster analysis identified different within-team synchronized groups. This strategy may help to identify particular sub-set of players and their specific coordination tendencies and roles during the game.
