# Correction: Competing with Lower Level Opponents Decreases Intra-Team Movement Synchronization and Time-Motion Demands during Pre-Season Soccer Matches

**DOI:** 10.1371/journal.pone.0120461

**Published:** 2015-03-16

**Authors:** The PLOS ONE Staff

The following information is missing from the Acknowledgements section: This study was part of a project registered at the Portuguese Foundation for Science (FCT, PEst-OE/SAU/UI4045/2014).
